# Intergeneration Bonding: An Acceptability and Feasibility Study

**DOI:** 10.1093/geroni/igab046.2644

**Published:** 2021-12-17

**Authors:** Lauren Rezac, Miechelle McKelvey, Ladan Ghazi Saidi

**Affiliations:** 1 University of Nebraska at Kearney, Kearney, Nebraska, United States; 2 University of Nebraska at Kearney, University of Nebraska at Kearney, Nebraska, United States

## Abstract

Social isolation and loneliness are detrimental risk factors to older adult’s physical and psychological well-being and quality of life. Given the current situation of the COVID-19 pandemic, social isolation has risen. Social isolation affects younger adults as well and may increase the chances of depression and anxiety. In this study, we tested the acceptability and feasibility of an Intergenerational Bonding (IGB) Program with older and younger adults. Participants include students aged 19-29 at the University of Nebraska at Kearney and older adults above the age of 60 residing in the community. First, we surveyed younger and older adults to determine their interest level in participating in an IGB Program. Both groups of participants rated their interest in different activities. The most popular activities among both groups included engaging in conversations, board games, and an exchange of skills. Neither of the groups was in favor of participating in free housing opportunities or sports-related activities. Response rates were high in young adults but low in older adults, due to lack of trust. Then, in a pilot study, we measured the feasibility and acceptability of the IGB Program. Older adults residing in independent dwellings, assisted living environments, nursing homes and members of community groups were invited to participate in the intergenerational program. Response rates of older adults were low. Further, establishing collaboration with institutions such as nursing homes was not easy despite initial interest. Building trust and working with community activity group facilitators would be beneficial in recruiting older adults.

